# Effects of Dietary Intake of Marine Ingredients on the Circulating Total Cholesterol Concentration in Domestic Dogs: A Systematic Review and Meta‐Analysis

**DOI:** 10.1111/jpn.14045

**Published:** 2024-09-18

**Authors:** Olivia Bysheim Helland, Linnea Vikane Andreassen, Anne Sofie Fischer, Oddrun Anita Gudbrandsen

**Affiliations:** ^1^ Department of Clinical Medicine Dietary Protein Research Group, Centre for Nutrition, University of Bergen Bergen Norway

**Keywords:** canine, *Canis familiaris*, cholesterol, long‐chain PUFA, pet food

## Abstract

A high circulating total cholesterol (TC) concentration increases the risk for atherosclerosis in the domestic dog. Intake of marine foods is associated with a lowering effect on circulating TC concentration in humans and rodents, but the reported effects of marine ingredients on the TC concentration in domestic dogs has not yet been reviewed. The main aim was to investigate the effects of consuming marine ingredients on the TC concentration in domestic dogs. A systematic literature search was performed using the databases PubMed, Web of Science and Embase, structured around the population (domestic dogs), intervention (source and type of marine ingredients, dose, duration), comparator (control diet) and the primary outcome (circulating TC). Articles were assessed for risk of bias using the SYRCLE's tool. A meta‐analysis was conducted in Review Manager v. 5.4.1 (the Cochrane Collaboration), comprising 12 articles with 243 dogs. Consumption of marine oils resulted in a significantly lower circulating TC concentration relative to comparator groups (mean difference −0.70 mmol/L, 95% CI (−1.21, −0.18), *p* = 0.008), with high statistical heterogeneity (*I* ^2^ = 78%). The risk of bias is unclear since few of the entries in the SYRCLE's tool were addressed. We did not identify any studies using marine proteins or marine organisms other that fish. To conclude, intake of marine oils results in a lower TC concentration in dogs, thus reducing an important risk factor for atherosclerosis in canines. This study was registered at www.crd.york.ac.uk/PROSPERO/ as CRD42023396943.

**Abbreviations**
CVD: cardiovascular disease
HDL‐C: HDL‐cholesterol
LDL‐C: LDL‐cholesterol
PUFA: polyunsaturated fatty acids
TC: total cholesterol

## 1 Introduction

Dogs are cholesteryl ester transfer protein deficient and therefore circulating cholesterol is mainly transported in HDL, making dogs less susceptible to develop atherogenesis compared to humans (Guyard‐Dangremont et al. 1998). Importantly, a high cholesterol concentration increases the risk for developing atherosclerosis in domestic dogs (Mahley et al. 1977). Excessive storage of fat in adipose tissues is associated with elevated cholesterol concentration and alterations in cardiac structure and function in dogs (Jeusette et al. 2005; Tropf et al. 2017; Peña et al. 2008; Park et al. 2014; Piantedosi et al. 2016). This is of concern since it was estimated that between 20% and 59% of domestic dogs were overweight or obese during the first two decades of the present millennium (Chandler et al. 2017). Atherosclerosis is rare in canines but has been documented in domestic dogs (Mahley et al. 1977; Geer 1965; Liu et al. 1986; Kagawa et al. 1998; Boynosky and Stokking 2014; Sottiaux 1999; Hess, Kass, and Winkle 2003; Hess, Kass, and Van Winkle 2006) and has been reported to coincide with hypercholesterolaemia (Mahley et al. 1977; Geer 1965; Liu et al. 1986; Kagawa et al. 1998; Boynosky and Stokking 2014; Sottiaux 1999; Hess, Kass, and Winkle 2003; Hess, Kass, and Van Winkle 2006), diabetes mellitus (Sottiaux 1999; Hess, Kass, and Winkle 2003; Hess, Kass, and Van Winkle 2006) or hypothyroidism (Liu et al. 1986; Hess, Kass, and Winkle 2003), however spontaneous atherosclerosis also occurs in dogs without indications of these endocrinopathies (Hess, Kass, and Winkle 2003; Hess, Kass, and Van Winkle 2006). Atherosclerosis that has developed secondary to high cholesterol concentration may be treated by weight loss and/or through changes in the diet.

Fish consumption is associated with beneficial health effects in humans, including a lower risk of cardiovascular diseases (CVDs) (Zheng et al. 2012; Virtanen et al. 2008; He et al. 2004). The beneficial effects of fish intake are assumed to be mainly caused by the n‐3 long‐chain polyunsaturated fatty acids (LC‐PUFA) eicosapentaenoic acid (EPA, C20:5n‐3) and docosahexaenoic acid (DHA, C22:6n‐3) (Mozaffarian and Wu 2011). Intake of fish has been associated with more beneficial cholesterol profile in some but not all clinical studies (Smith et al. 2009; Telle‐Hansen et al. 2012; Mori et al. 1999; Gunnarsdottir et al. 2008; Lindqvist et al. 2009; Lara et al. 2007; Hagen et al. 2016; Bratlie et al. 2021), and a recent systematic review with meta‐analysis showed that fish protein intake prevented high total cholesterol (TC) concentration in rodents (O'Keeffe and Gudbrandsen 2023). Therefore, the main aim of this systematic review and meta‐analysis was to investigate the effect of consumption of marine ingredients on the circulating TC concentration in domestic dogs. The secondary aim was to provide a narrative synthesis of the findings from the included articles structured around the population (domestic dogs), the intervention (description of the diet including source and type of marine ingredients, dose, duration) and the comparator diet (description of the control diet) on the circulating concentrations of TC, HDL‐cholesterol (HDL‐C) and LDL‐cholesterol (LDL‐C). Findings from this systematic review and meta‐analysis will provide important knowledge regarding the possible health effects of marine ingredients for dogs and may be important for the design of future studies targeting the effects of marine ingredients on cholesterol metabolism in dogs.

## 2 Methods

### 2.1 Animal Welfare Statement

This manuscript does not include original research data.

### 2.2 Protocol and Registration

The review protocol can be viewed at the International Prospective Register of Systematic Reviews (PROSPERO) website (https://www.crd.york.ac.UK/PROSPERO) with registration number CRD42023396943.

### 2.3 Search Strategy

A comprehensive literature search was carried out in accordance with the Preferred Reporting Items for Systematic Reviews and Meta‐Analyses (PRISMA) guidelines (Page et al. 2021). We used the electronic databases PubMed (https://pubmed.ncbi.nlm.nih.gov/), Web of Science (https://clarivate.com/products/web-ofscience/) and Embase (http://www.elsevier.com/online-tools/embase). The four authors (O.B.H., L.V.A., A.S.F., O.A.G.) conducted the search independently in June 2023, using a combination of the following search terms to identify relevant articles on the effects of marine ingredients on serum/plasma cholesterol concentration in domestic dogs: ((abalone) OR (algae) OR (anchovy) OR (anglerfish) OR (bass) OR (billfish) OR (bivalvia) OR (bonito) OR (calanus) OR (capelin) OR (catfish) OR (cephalopoda) OR (clam) OR (cockle) OR (cod) OR (codlings) OR (copepod) OR (crab) OR (crustacea) OR (cusk) OR (dogfish) OR (dorado) OR (eel) OR (fish*) OR (flatfish) OR (flounder) OR (fur seal) OR (grouper) OR (haddock) OR (hake) OR (hakelings) OR (halibut) OR (herring) OR (kelp) OR (krill) OR (labridae) OR (ling) OR (lobster) OR (mackerel) OR (marlin) OR (menhaden) OR (mora) OR (mussel) OR (octopus) OR (oyster) OR (plaice) OR (pompano) OR (pollock) OR (redfish) OR (rocklings) OR (saithe) OR (salmon) OR (sardine) OR (saury) OR (seafood) OR (sea food) OR (sea lion*) OR (sea shell) OR (seaweed) OR (sea weed) OR (seal*) OR (sea trout) OR (scallop) OR (shark) OR (shrimp) OR (smooth‐hound) OR (snails) OR (snapper) OR (sole fish) OR (sprat) OR (squid) OR (swordfish) OR (tuna) OR (turbot) OR (tusk) OR (walrus) OR (whelk) OR (whale) OR (whiting) OR (wolffish) OR (wrasse)) AND ((dog) OR (dogs) OR (canine) OR (canines) OR (hound) OR (hounds)) AND ((diet) OR (diets) OR (dietary) OR (food) OR (foods) OR (feed) OR (fodder) OR (meal) OR (meals) OR (supplement) OR (supplements) OR (supplementation) OR (supplementations) OR (animal feed)) AND ((cholesterol) OR (HDL) OR (LDL) OR (lipoprotein)). The reference lists of the reviewed articles were checked manually to identify relevant studies, and PubMed, Web of Science, Embase and PROSPERO were searched to avoid overlap with existing systematic reviews. A supplementary literature search conducted on 8 November 2023 identified one relevant publication since the first search.

### 2.4 Selection Criteria

We based the search strategy on the PICO (population, interventions, comparisons and outcomes) framework (Richardson et al. 1995). The eligibility criteria were (1) population: only articles from intervention studies using adult domestic dogs (*Canis familiaris*) were included, (2) intervention: the interventions must include marine ingredients either as part of a diet or as a supplement for a period of more than 5 days, thus excluding marine ingredients administered in drinking water or by injection, single‐dose administration and short time studies (≤ 5 days), (3) comparison: the study design must include a control group fed a relevant control diet, and (4) outcomes: the main outcome was circulating TC concentration, the secondary outcomes were HDL‐C and LDL‐C concentrations. Review articles, protocols, abstracts, posters and grey literature were not included. We did not exclude articles on the basis of the articles' publication year during the identification process, and articles were manually removed if there were no English full texts available.

The search and the evaluation of the articles were performed independently by the four authors. The retrieved articles from PubMed, Web of Science and Embase were collected in the free web tool Rayyan (https://www.rayyan.ai), where duplicates were identified by the software and then removed manually. The initial screening was based on the title and abstract, followed by a full‐text screening of the eligible publications for final inclusion. All authors independently assessed each article and any discrepancies were resolved through discussion, and articles were included or excluded based on the eligibility criteria.

**Figure 1:**
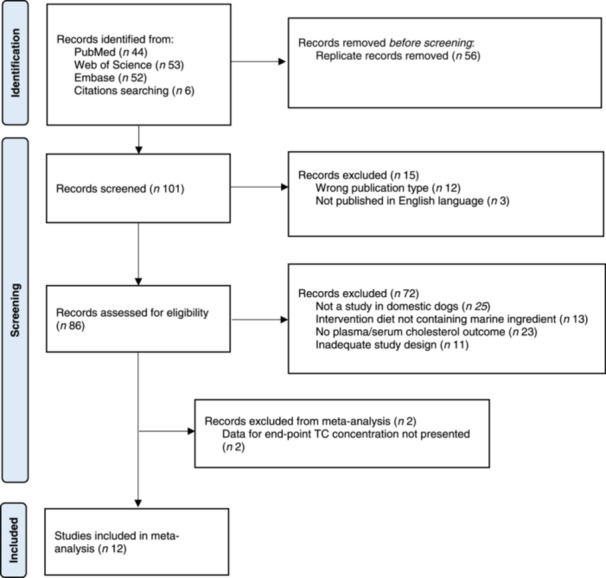
PRISMA flowchart of literature search via databases, showing the selection of studies for inclusion in the systematic review and meta‐analysis. [Color figure can be viewed at wileyonlinelibrary.com]

The PRISMA 2020 flow diagram (Page et al. 2021) gives an overview of the selection process (Figure 1). In total, 14 articles were found eligible and were included in this systematic review, whereas 12 articles were included in the meta‐analysis.

### 2.5 Data Extraction and Criteria Appraisal

Data were extracted from article texts, tables and figures based on the categories defined for each of the PICO categories as described above. The data extracted from the articles included dog breed, sex, age, bodyweight, body condition score (BCS) and circulating TC concentration at baseline, description of the intervention and control groups, number of dogs per group, description of the intervention diet and the comparator diet, the duration of the intervention period and the housing condition. Data collected for outcome measurements comprised the main outcome, that is, circulating TC concentration (described in all included articles), and the secondary outcomes were concentrations of HDL‐C and LDL‐C. Information on any changes in bodyweight is narratively described.

The results were defined as statistically significant where *p* < 0.05. For articles that included multiple dietary groups, only data from the relevant groups were included in this review, that is, groups consuming marine ingredients and their relevant control group. The data extraction was completed independently by all authors. When inconsistencies were observed in the articles, for example, missing data or information, or suspicion of possible errors, the corresponding author of the article in question was contacted by e‐mail.

The study designs in the included articles were highly heterogeneous, therefore, we include a descriptive approach in addition to a meta‐analysis.

### 2.6 Assessments of Risk of Bias and Study Quality

The SYRCLE's risk of bias tool (Hooijmans et al. 2014), which is an adapted version of Cochrane's risk of bias tool for clinical randomised trials (Higgins et al. 2011), was used to assess for risk of bias in the reviewed articles. The 10 entries in the SYRCLE tool, with detailed signalling questions to help uncover potential bias, were answered with Yes (low risk of bias), No (high risk of bias) or Unclear (unclear risk of bias). The quality of the included articles was evaluated by using a combination of the CAMARADES checklist (Macleod et al. 2004) and items from the ARRIVE 2.0 guidelines (Percie du Sert et al. 2020). We selected these items to incorporate matters subjectively viewed as necessary for evaluating the overall quality of the studies, and we avoided items already covered by the SYRCLE's risk of bias tool. The articles were scored with one point for reporting the required information and zero points when information was missing, with a total score of maximum 10 points, with 1–3 points regarded as low quality, 4–7 points regarded as medium quality and 8–10 points regarded as high quality. All authors independently assessed each article for risk of bias and study quality, and any discrepancies were resolved through discussion.

### 2.7 Statistical Analyses

The effect of consuming diets with marine ingredients on the circulating TC concentration from 12 of the identified articles (LeBlanc et al. 2005; Hall et al. 2011, 2002; Boretti et al. 2019; Brown et al. 1998, 2000; Pellegrino et al. 2021; Barrouin‐Melo et al. 2016; Wander et al. 1997; Kearns et al. 1999; Landymore et al. 1985, 1986) was meta‐analysed using Review Manager v. 5.4.1 (The Nordic Cochrane Centre 2014) (provided by the Cochrane Collaboration), with comparison to control diets. The mean and standard deviation for endpoint TC concentration and the number of dogs were registered for intervention and comparator groups. Standard deviation was calculated from the standard error of the mean when not provided in the articles. In articles presenting the TC concentration using graphs, we did our best to estimate each group's mean and spread. TC concentrations presented as mg/dL were converted to mmol/L by multiplying with 0.02586. Data were treated as continuous measures, and the intervention and comparator groups were compared using the random effects inverse‐variance model, and the statistical heterogeneity between the studies was evaluated and is expressed as measures of Cochran's *Q* (*χ* ^2^ test) and *I* ^2^. Sensitivity analyses were conducted through a leave‐one‐out analysis to evaluate the effect of confounding factors on the robustness; the comparisons with the highest positive and negative effect sizes and the studies with the highest risk of bias or lowest quality of evidence were excluded sequentially from the meta‐analysis. The publication bias was evaluated by using a funnel plot. The result from the meta‐analysis was visualised as a forest plot. *p* < 0.05 was considered statistically significant. The secondary outcomes are reviewed narratively.

## 3 Results

### 3.1 Search Results and Study Characteristics

One‐hundred and one potentially relevant articles were identified through searches in the electronic databases and the reference lists of the reviewed articles that were checked manually. Of these, 15 were excluded (12 were the wrong publication type and three were not published in English language). Eighty‐six articles were assessed for eligibility, and of these, 39 were excluded based on the title or abstract, and 33 were excluded after full‐text screening. Thus, a total of 14 articles met our defined PICO criteria (LeBlanc et al. 2005; Hall et al. 2011, 2002; Boretti et al. 2019; Brown et al. 1998, 2000; Pellegrino et al. 2021; Barrouin‐Melo et al. 2016; Wander et al. 1997; Kearns et al. 1999; Landymore et al. 1985, 1986; Smith et al. 2007; Jackson and Jewell 2023) and were included in this systematic review (Figure 1). Two articles did not provide the necessary information for the meta‐analysis (Smith et al. 2007; Jackson and Jewell 2023) and are therefore only narratively described.

The screening process returned no research on marine products other than oils on the effect on TC concentration in domestic dogs. We did not identify any studies using marine organisms other than fish, or marine proteins, and the included studies were therefore limited to marine oil as the intervention.

The articles were published between the years 1985 and 2023 and comprised a total of 146 dogs in the intervention group and 145 dogs in the comparator group. Seven articles included both male and female dogs (LeBlanc et al. 2005; Hall et al. 2011; Boretti et al. 2019; Brown et al. 1998, 2000; Barrouin‐Melo et al. 2016; Jackson and Jewell 2023), whereas two studies used only female dogs (Hall et al. 2002; Wander et al. 1997), one study used only male dogs (Pellegrino et al. 2021) and four studies did not declare the sex of the included dogs (Kearns et al. 1999; Landymore et al. 1985, 1986; Smith et al. 2007). Different dog breeds and mixed breeds were used in the studies; solely beagles (Hall et al. 2011, 2002; Boretti et al. 2019; Wander et al. 1997), boxer dogs (Smith et al. 2007), Walker hound‐cross dogs (LeBlanc et al. 2005) or mixed‐breed dogs (Brown et al. 1998, 2000; Pellegrino et al. 2021; Barrouin‐Melo et al. 2016; Landymore et al. 1985, 1986; Jackson and Jewell 2023) and different breeds (Pellegrino et al. 2021), beagles and mixed‐breed dogs (Jackson and Jewell 2023), or Labrador Retrievers and Fox Terriers (Kearns et al. 1999).

The dogs were housed at research facilities (LeBlanc et al. 2005; Hall et al. 2011, 2002; Boretti et al. 2019; Brown et al. 1998, 2000; Wander et al. 1997; Kearns et al. 1999; Landymore et al. 1985, 1986; Jackson and Jewell 2023) or were housed with their owners (Pellegrino et al. 2021; Barrouin‐Melo et al. 2016; Smith et al. 2007). The intervention periods lasted from 28 days to 20 months, and the study sizes were between 9 and 71 dogs. The dogs in the included studies were described as being adult (Brown et al. 1998, 2000; Landymore et al. 1985, 1986), geriatric (Hall et al. 2002; Wander et al. 1997), or age 1 year or older (LeBlanc et al. 2005; Hall et al. 2011; Boretti et al. 2019; Pellegrino et al. 2021; Barrouin‐Melo et al. 2016; Smith et al. 2007; Jackson and Jewell 2023) at the start of intervention. Kearns et al. (1999) used both old and young adult dogs. The dogs were considered healthy in seven of the studies (LeBlanc et al. 2005; Hall et al. 2011, 2002; Boretti et al. 2019; Pellegrino et al. 2021; Wander et al. 1997; Jackson and Jewell 2023). Two of the studies used dogs that had undergone a partial nephrectomy (Brown et al. 1998, 2000), one study used dogs with arrhythmogenic right ventricular cardiomyopathy (Smith et al. 2007), one study used dogs with autogenous vein implantation (Landymore et al. 1985), one study used dogs with autologous vein implantation (Landymore et al. 1986) and one study used dogs with canine spontaneous osteoarthritis (Barrouin‐Melo et al. 2016). One article did not describe the health status of the dogs (Kearns et al. 1999). The TC concentration in serum/plasma at baseline was presented separately for the intervention group and the comparator group in eight articles (LeBlanc et al. 2005; Hall et al. 2011; Boretti et al. 2019; Brown et al. 1998, 2000; Pellegrino et al. 2021; Barrouin‐Melo et al. 2016; Smith et al. 2007), combined for intervention and comparator groups in two articles (Landymore et al. 1985, 1986), and was not provided in four articles (Hall et al. 2002; Wander et al. 1997; Kearns et al. 1999; Jackson and Jewell 2023). All articles reported that the mean TC concentration was within the normal reference range for dogs (3.49−7.19 mmol/L) at baseline (Krimer 2011), however, some of the dogs in the studies by Smith et al. (2007) and Barrouin‐Melo et al. (2016) had TC concentration above the reference range at baseline. The study characteristics are presented in Table 1.

**Table 1:** Study characteristics and outcomes.

First author (year)	Description of dog breeds, health condition and housing situation	Brief description of the experimental diets (including content of n‐3 LC‐PUFAs if provided) and name of product/producer, duration of interventions	Description of the experimental groups (the number of dogs [*n*], sex, age, BW, BCS and circulating total cholesterol [TC] concentration at baseline/start of intervention)	Total cholesterol (TC), HDL‐cholesterol (HDL‐C) and LDL‐cholesterol (LDL‐C) concentrations in serum/plasma at end of study in dogs fed a diet containing marine ingredients compared with control group
Landymore et al. (1985)	Mixed‐breed dogs with autogenous vein implantation. Housed at a research facility.	No description of a pre‐study diet. Intervention diet, 7 weeks: A 2% cholesterol diet (I.C.N Nutritional Biochemicals, Cleveland, OH) supplemented with cod‐liver oil capsules (1.8 g EPA daily, DHA N/A). Control diet, 7 weeks: A 2% cholesterol diet (I.C.N Nutritional Biochemicals) without any supplement. EPA, DHA: N/A.	Age and BW presented for all included dogs (*n* 14) Age: Adult (not further specified) BW: Range 25−30 kg BCS and sex: N/A TC: Mean 4.6 (SD 0.4) mmol/L Intervention group (*n* 7) Control group (*n* 7) Information on sex distribution, age, BW, BCS and TC for individual groups were not provided	TC: Lower in intervention group HDL‐C and LDL‐C: N/A
Landymore et al. (1986)	Mixed‐breed dogs with autologous vein implantation. Housed at a research facility.	No description of a pre‐study diet. Intervention diet, 7 weeks: A 2% cholesterol diet (I.C.N Nutritional Biochemicals, Cleveland, OH) supplemented with cod‐liver oil capsules (1.8 g EPA daily, DHA N/A). Control diet, 7 weeks: A 2% cholesterol diet (I.C.N Nutritional Biochemicals) without any supplement. EPA, DHA: N/A.	Age and BW presented for 24, included dogs Age: Adult (not further specified) BW: Range 26−30 kg BCS and sex: N/A TC: Mean 4.5 (SD 0.2) mmol/L Intervention group (*n* 8) Control group (*n* 8) Information on sex distribution, age, BW, BCS and TC for individual groups were not provided	TC: Lower in intervention group HDL‐C and LDL‐C: N/A
Wander et al. (1997)	Beagles, healthy. Housed at a research facility.	Pre‐study diet, duration not specified: A commercial diet (Science Diet Canine Maintenance, Hill's Pet Nutrition, Topeka, KS), low n‐3:n‐6 ratio. Intervention diet, 8 weeks: An experimental diet (Hill's Pet Nutrition) containing 2% fish oil (not specified, from Zapata Protein, Reedville, VA). 3.0 g EPA and 2.65 g DHA per kg diet. Control diet, 8 weeks: An experimental diet (Hill's Pet Nutrition) containing 2% corn oil (Mazola, Englewood Cliffs, NJ) < 0.1 g EPA and < 0.1 g DHA per kg diet.	Intervention group (*n* 7) 7 females Age: Geriatric, mean 10.1 (SEM 0.37) years BW: Mean 11.5 (SEM 0.9) kg BCS: N/A TC: N/A Control group (*n* 6) 6 females Age: Geriatric, mean 10.2 (SEM 0.11) years BW mean 12.5 (SEM 1.1) kg BCS N/A TC: N/A	TC and HDL‐C: Lower in intervention group LDL‐C: N/A
Brown et al. (1998)	Mixed breed dogs (not further specified) with renal insufficiency. Housed at a research facility.	Pre‐study diet, 2 months: A commercial diet (Hill's Science Diet, Hill's Petfoods, Topeka, KS), EPA and DHA contents N/A. Intervention diet, 20 months: An experimental diet (Mark Morris Associates, Topeka, KS) with added 15 wt% of dog's energy intake as menhaden oil (Zapata‐Haynie Menhaden Oil Refinery, Reedville, VA). The fish oil contained 13.6% EPA and 12.5% DHA. Control diet, 20 months: An experimental diet (Mark Morris Associates, Topeka, KS) with added 15 wt% of dog's energy‐intake as safflower oil (Oil Seeds International, San Francisco, CA). Safflower oil contained 77.7% LA.	Intervention group (*n* 7): 4 males, 3 females Age: Adult (not further specified) BW: Mean 12.3 (SEM 1.4) kg BCS: N/A TC: Mean 5.56 (SD 1.09) mmol/L Control group (*n* 7): 4 males, 3 females Age: Adult (not further specified) BW mean 11.4 (SEM 1.1) kg BCS: N/A TC: Mean 5.46 (SD 1.23) mmol/L	TC and HDL‐C: Lower in the intervention group LDL‐C: N/A
Kearns et al. (1999)	Two experiments: Labrador Retrievers and Fox Terriers, described as old adults or young adults. Health condition N/A, Housed at a research facility.	Pre‐study diet, 60 days: An experimental diet with high n‐6:n‐3 ratio (25:1), containing 8.21% chicken fat and no fish oil. Intervention diet, 60 days: The same diet as the pre‐study diet, with 1.65% fish oil (not specified) and 6.50% chicken fat Control diet, 60 days: The same diet as the pre‐study diet.	Old adults: 9 Labrador Retrievers, mean age 9.6 years; 9 Fox Terriers, mean age 11.5 years Young adults: 9 Labrador Retrievers, mean age 1.5 years; 9 Fox Terriers, mean age 1.8 years Nine dogs in each group were given the intervention diet, the other nine continued on the pre‐study diet (control diet) Information on sex distribution, age, BW, BCS and TC for individual groups were not provided	Old adults: TC: Higher in intervention group HDL‐C and LDL‐C: N/A Young adults: TC: NS HDL‐C and LDL‐C: N/A
Brown et al. (2000)	Mixed breed dogs (not further specified), with renal insufficiency Housed at a research facility.	Pre‐study diet, 2 months: A commercial diet (Hill's Science Diet, Hill's Petfoods, Topeka, KS), EPA and DHA contents not specified. Intervention diet, 10−13 weeks: An experimental diet (Mark Morris Associates, Topeka, KS) + 15 wt% of dog's intake with menhaden oil (Zapata‐Haynie Menhaden Oil Refinery, Reedville, VA). Fish oil: 13.6% EPA, 12.5% DHA. Control diet, 10−13 weeks: An experimental diet (Mark Morris Associates, Topeka, KS) + 15 wt% of dogs' energy‐intake with safflower oil (Oil Seeds International, San Francisco, CA), Safflower oil: 77.7% linoleic acid.	Intervention group (*n* 6): 3 males, 3 females Age: Adult (not further specified) BW: Mean 15.5 (SEM 2.5) kg BCS: N/A TC: Mean 5.17 (SD 0.63) mmol/L Control group (*n* 6): 3 males, 3 females Age: Adult (not further specified) BW: Mean 16.0 (SEM 2.1) kg BCS: N/A TC: Mean 5.04 (SD 1.46) mmol/L	TC and HDL‐cholesterol: NS LDL‐cholesterol: N/A
Hall et al. (2002)	Beagles, healthy. Housed at a research facility.	Pre‐study diet, 90 days: A commercial diet (Science Diet Canine Maintenance, Hill's Pet Nutrition Inc., Topeka, Kan). (n‐6 to n‐3 ratio 18:1. n‐3 source 90% plant‐derived from soybean oil (a‐linolenic acid). Intervention diet, 82 days: A commercial diet (Science Diet Canine Maintenance, Hill's Pet Nutrition Inc.) with added 2% menhaden oil (Zapata Protein, Reedville, VA) EPA 1.9 g/kg diet, DHA 2.5 g/kg diet. Control diet, 82 days: A commercial diet (Science Diet Canine Maintenance, Hill's Pet Nutrition Inc.) with added 2% corn oil (Mazola corn oil, Mazola, Englewood Cliffs, NJ), EPA < 0.1 g/kg diet, DHA < 0.1 g/kg diet).	Age and BW presented for all included dogs: Age: Geriatric (7−10 years) BW: Range 8.1−15.9 kg BCS: N/A TC: N/A Intervention group (*n* 6) 6 females Control group (*n* 6) 6 females Information on age, BW, BCS and TC for individual groups were not provided	TC: Lower in intervention group HDL‐C and LDL‐C: N/A
LeBlanc et al. (2005)	Walker hound‐cross dogs, healthy. Housed at a research facility.	Pre‐study diet, for a period of months to years (since entry into the colony as an adult: A commercial diet (Laboratory Canine Diet 5006, Lab Diet, Purina Mills, Gray Summit, MO), contained < 0.1 g EPA and 0.1 g DHA per kg diet. Intervention diet, 12 weeks: 500 g of Laboratory Canine Diet (Lab Diet, Purina Mills) added 0.6 g of sunflower oil, 7 g of menhaden oil (1.65% oil on dry‐matter basis; Omega Protein, Reedville, VA), and a 3−5 g compressed portion of Prescription Diet Canine r/d (Hill's Pet Nutrition, Topeka, KS), 1.7 g EPA, 2.2 g DHA per kg diet. Control diet, 12 weeks: 500 g of Laboratory Canine Diet (Lab Diet, Purina Mills), added 12.4 g of sunflower oil and a 3−5 g compressed portion of Prescription Diet Canine r/d (Hill's Pet Nutrition), < 0.1 EPA and 0.1 DHA per kg diet.	Age, BW and BCS presented for 15 included dogs: 8 females, 7 males Age: Range 1−4 years BW: Range 21−27 kg BCS (1−9): Range 5−6 Intervention group (*n* 5) TC: Mean 4.38 (SD 1.04) mmol/L Control group (*n* 5) TC: Mean 4.32 (SD 1.45) mmol/L Information on sex distribution, age, BW and BCS for individual groups were not provided	TC: NS LDL‐C and HDL‐C: N/A
Smith et al. (2007)	Boxer dogs with arrhythmogenic right ventricular cardiomyopathy. Housed with owners.	Pre‐study diet: The dogs' normal feed, must correspond to < 1.5 g of total n‐3 PUFA per day. Intervention diet, 6 weeks: The dogs' normal feed, must contain < 1.5 g of total n‐3 PUFA per day, with added two capsules per day of fish oil (not specified) containing 390.0 mg EPA, 248.5 mg DHA. Control diet, 6 weeks: The dogs' normal feed, must contain < 1.5 g of total n‐3 PUFA per day, with added two capsules per day of sunflower oil, containing no EPA or DHA.	Intervention group (*n* 8) Sex: N/A Age: Median 6.1 (range 4.2−9.3) years BW: Median 28.0 (range 23.6−35.6) kg BCS (1−9): Median 5.75 (range 5.0−7.0) TC: Median 5.79 (range 4.99−7.27) mmol/L Control group (*n* 8) Sex: N/A Age: Median 6.6 (range 4.5–9.3) years BW: Median 30.6 (range 23.6−40.2) kg BCS (1−9): Median 6.0 (range 5.0−7.5) TC: Median 6.36 (range 4.60−7.45) mmol/L	TC: Change from baseline to endpoint similar in intervention group and control group LDL‐C and HDL‐C: N/A Concentrations at endpoint were not presented, this study is not included in the meta‐analysis
Hall et al. (2011)	Beagles, healthy. Housed at a research facility.	Pre‐study diet, 60 days: A complete and balanced food (Hill's Pet Nutrition Inc.) containing 0.01% EPA and 0.00% DHA. Intervention diet, 90 days: An experimental food with added 1.35% fish oil (not specified, from Hill's Pet Nutrition Inc.), containing 0.25% EPA and 0.17% DHA of diet. Control diet, 90 days: An experimental food with no addition of fish oil (Hill's Pet Nutrition Inc.), containing 0.01% EPA, 0.01% DHA of diet (no information on lipid source provided).	Age, BW and BCS presented for all included dogs: 25 males, 25 females Age: Mean 5.3 (range 1.4−14.2) years BW: Mean 12.0 (SEM 0.4) kg BCS: N/A Intervention group (*n* 10) TC: Mean 6.88 (SD 0.61) mmol/L Control group (*n* 10) TC: Mean 4.05 (SD 0.28) mmol/L Information on sex distribution, age, BW and BCS for individual groups were not provided	TC: NS HDL‐C and LDL‐C: N/A
Barrouin‐Melo et al. (2016)	Various mixed‐breed dogs and different breeds^a^ with canine spontaneous osteoarthritis. Housed with owners.	Pre‐study diet, 2 weeks: Commercial diets containing wheat/beef (Royal Canin Croc) or rice/chicken sensitive formula (Jahti & Vahti lamb and rice), did not contain fish oil derived n‐3 PUFAs. Intervention diet, 16 weeks: The same diet as the pre‐study diet, with added 0.2 mL of a concentrated oil product from deep sea fish per kg BW (Doils joints; Nutraceuticoils, Belgium). 110.25 (SD 5.75) mg n‐3 PUFA (predominantly EPA and DHA) per kg BW.^c^ Control diet, 16 weeks: The same pre‐study diet with added 0.2 mL of corn oil per kg BW (Doils joints; Nutraceuticoils). 1.7 (SD 0.09) mg of n‐3 PUFA (predominantly ALA) per kg BW.^c^	Age, BW and BCS presented for all 77 included dogs^c^ Intervention group (*n* 35 of 39): 17 males, 22 females Age: Mean 5.6 (SD 2.9) years BW: Mean 33.5 (SD 12.0) kg BCS: Median 3 (range 2−5) TC: 6.51 ± 1.90 mmol/L^d^ Control group (*n* 36 of 38): 17 males, 21 females Age: Mean 6.5 (SD 3.0) years BW: Mean 34.2 (SD 8.6) kg BCS: Median 3 (range 2−4) TC: 7.13 ± 1.62 mmol/L^d^	TC: NS HDL‐C and LDL‐C: N/A
Boretti et al. (2019)	Beagles, healthy. Housed at a research facility.	Pre‐study diet, ≥ 3 months: A commercial diet (JOSIdog adult sensitive, Josera petfood GmbH & Co. KG, Kleinheubach, Germany) containing 0% EPA & DHA on dry matter basis. Intervention diet, 3 months: A self‐made diet enriched with linseed and salmon oil, containing 0.31% EPA, 0.44% DHA on dry matter basis. Control diet, 3 months: Commercial diet (Purina Pro Plan Performance, Purina, Nestlé Purina PetCare, Vevey, Switzerland) containing 0.14% EPA, 0.06% DHA on dry matter basis.	Age, BW and BCS presented for all included dogs: Age: Median 4 (range 4−6.5) years BW: Median 13.8 (range 9−16.3) kg BCS: All dogs had 4 of 9 Intervention group (*n* 8): 4 males, 4 females TC: Mean 5.2 (SD 1.7) mmol/L Control group (*n* 8): 4 males, 4 females TC: Mean 5.3 (SD 0.9) mmol/L Information on age, BW and BCS for individual groups were not provided	TC: Lower in the intervention group. HDL‐C and LDL‐C: N/A
Pellegrino et al. (2021)	Various mixed‐breed dogs and different breeds^b^, healthy. Housed with owners.	Pre‐study diet, 4 weeks: A commercial diet (provided by Vitalcan, Argentina), containing 0.1% EPA and 0.2% DHA. Intervention diet, 90 days: The same commercial diet as the pre‐study diet (Vitalcan) supplemented with one capsule of 1000 mg fish oil (containing 23.2% EPA, 13.6% DHA (type of oil not specified). Control diet, 90 days: The same commercial diet as the pre‐study diet (Vitalcan) (0.1% EPA, 0.2% DHA), no supplementation.	Age, BW and BCS presented for all included dogs: Age: Mean 6.2 (SD 1.5, range 5−8) years BW: Mean 30.2 (SD 3.0, range 25−35) kg BCS: 3 of 5 Intervention group (*n* 5): 5 males TC: Mean 5.9 (SD 1.1) mmol/L Control group (*n* 4): 4 males TC: Mean 5.6 (SD 1.1) mmol/L Information on age, BW and BCS for individual groups were not provided	TC and LDL‐C: NS HDL‐C: Lower in intervention group
Jackson and Jewell (2023)	Beagles and mixed‐breed dogs, healthy. Housed at a research facility.	Pre‐study diet, 14 days: Experimental diet 0% EPA, 0.02% DHA. Intervention diet, 28 days: Experimental diet with 2.85 wt% added MEG‐3 0355TG fish oil (DSM Inc., Parsippany, NJ, USA), 11% pork fat. 0.18% EPA, 1.3% DHA in diet. Control diet, 28 days: Same as pre‐study diet, 13.9% pork fat.	Intervention group (*n* 16) 15 beagles, 1 mixed‐breed 8 females, 8 males Age: Mean 5.7 (SD 4.1) years BW: Mean 12.2 (SD 4.0) kg BCS: N/A TC: N/A Control group (*n* 16) 15 beagles, 1 mixed‐breed 8 females, 8 males Age: Mean 5.7 (SD 3.9) years BW: Mean 11.8 (SD 3.4) kg BCS: N/A TC: N/A	TC: Larger reduction from baseline to endpoint in intervention group HDL‐C and LDL‐C: N/A Concentrations at endpoint were not presented, this study is not included in the meta‐analysis

Abbreviations: BCS, body condition score; BW, bodyweight; HDL‐C, HDL‐cholesterol; LDL‐C, LDL‐cholesterol; N/A, data not available; NS, not statistically significant; TC, total cholesterol.

^a^ Collies, German Shepherd, Golden Retrievers, Labradors, Baucerons, Bernen Sennen Dogs, Rottweilers and mixed‐breed dogs.

^b^ Weimaraner, Boxer dogs and mixed‐breed dogs.

^c^ Information from Hielm‐Björkman et al. (2012).

d Did not describe if the spread was standard deviation or standard error of the mean.

### 3.2 Assessments of Risk of Bias

The risk of bias in the reviewed articles was assessed by using the SYRCLE's risk of bias tool for animal studies (Hooijmans et al. 2014) (Table S1). The use of sequence generation (#1) for reducing selection bias was described in three of the included articles, which specified that the dogs were allocated to the experimental groups by use of computer‐generated randomisation lists (Pellegrino et al. 2021; Barrouin‐Melo et al. 2016; Smith et al. 2007) and these articles were graded with ‘Yes’. Six of the articles stated that the dogs were randomly allocated to groups but provided no description of the allocation process (LeBlanc et al. 2005; Hall et al. 2011; Boretti et al. 2019; Brown et al. 1998, 2000; Jackson and Jewell 2023) and were graded with ‘Unclear’. Two articles ranked the dog on the basis of bodyweight for assignment to experimental groups (Hall et al. 2002; Wander et al. 1997) and were graded with ‘No’, and three articles did not describe the allocation process (Kearns et al. 1999; Landymore et al. 1985, 1986) and were graded with ‘Unclear’.

The majority of the reviewed articles included a comparison of the experimental groups at baseline and were graded with ‘Yes’ for reporting similar baseline characteristics between the groups (#2). These articles stated that groups were similar with regard to bodyweight (LeBlanc et al. 2005; Hall et al. 2002; Brown et al. 1998, 2000; Wander et al. 1997; Smith et al. 2007; Jackson and Jewell 2023), age (Hall et al. 2011; Smith et al. 2007; Jackson and Jewell 2023), BCS (Smith et al. 2007) and/or TC concentration (LeBlanc et al. 2005; Boretti et al. 2019; Brown et al. 1998, 2000; Wander et al. 1997). The between‐group sex distribution was stated to be similar in three articles (Hall et al. 2011; Boretti et al. 2019; Jackson and Jewell 2023), and three articles used only male dogs (Pellegrino et al. 2021) or only female dogs (Hall et al. 2002; Wander et al. 1997). One article described that the experimental groups were matched by age, sex (all male), bodyweight and body condition but did not provide a statistical comparison of these parameters (Pellegrino et al. 2021). The serum TC concentration was lower in the fish oil group compared to the comparator group in one study, but the results were not adjusted, possibly causing a minor increased risk of bias (Smith et al. 2007). However, we considered the risk for selection bias to be low. In the articles by Landymore et al. (1985, 1986) and Barrouin‐Melo et al. (2016), baseline data were presented for all included dogs and not solely for the dogs that completed the study, and we, therefore, considered the risk for selection bias to be unclear. The study by Kearns et al. (1999) did not present any comparisons of the experimental groups at baseline. Measures for allocation concealment (#3) were not described in any of the reviewed articles, thus all articles were graded with an ‘Unclear’ risk of bias. The dogs were either housed at a research facility (LeBlanc et al. 2005; Hall et al. 2011, 2002; Boretti et al. 2019; Brown et al. 1998, 2000; Wander et al. 1997; Kearns et al. 1999; Landymore et al. 1985, 1986; Jackson and Jewell 2023) or lived with their owners (Pellegrino et al. 2021; Barrouin‐Melo et al. 2016; Smith et al. 2007), and information on whether random housing (#4) was used was not provided in any of the articles. Since we consider that it is unlikely that the outcome measurement was influenced by not randomly housing the animals (the signalling question for #4), all articles received ‘Yes’, that is, a low risk of bias.

The use of double‐blinding of both caregivers and investigators were described in three articles (Barrouin‐Melo et al. 2016; Smith et al. 2007; Jackson and Jewell 2023), and were scored with ‘Yes’ for the use of blinding in relation to performance bias (#5), whereas the rest of the articles did not provide relevant information and were graded with ‘Unclear’. In three articles, the dogs' caregivers were not blinded as the intervention group were supplemented with fish oil capsules, whereas the comparator group did not receive a placebo supplement (Pellegrino et al. 2021; Landymore et al. 1985, 1986), therefore these articles were graded with ‘No’ symbolising a high risk of performance bias. The detection bias was unclear in 11 articles (LeBlanc et al. 2005; Hall et al. 2011, 2002; Boretti et al. 2019; Brown et al. 1998, 2000; Wander et al. 1997; Kearns et al. 1999; Landymore et al. 1985, 1986; Smith et al. 2007), with only three articles stating that the outcome assessors were blinded and were graded with ‘Yes’ (Pellegrino et al. 2021; Barrouin‐Melo et al. 2016; Jackson and Jewell 2023) for a low risk of detection bias (#7). All articles were graded as having an unclear risk of bias for the random outcome assessment (#6) and the selective outcome reporting (#9), and all articles received ‘Yes’, that is, a low risk of bias when scoring the articles for incomplete outcome data (#8) as all the expected data were included.

With regard to the last item on the list, that is, other sources of bias (#10), we chose to focus on any donations from producers of pet food or ingredients for pet food, and whether authors were employed by such companies, and other potential risks especially associated with the dogs that lived with their owner. Feed for the interventions were donated by the pet food producers Hill's Science and Technology Center and Hill's Petfoods (Brown et al. 1998, 2000), Vitalcan (Pellegrino et al. 2021), and other articles stated that the studies were supported in part by Hill's Pet Nutrition Inc. (Hall et al. 2011, 2002; Wander et al. 1997; Jackson and Jewell 2023), Royal Canin (Smith et al. 2007), the VCS Corp Fund (not further specified) (LeBlanc et al. 2005) or the fish oil manufacturing company Nutriceuticoils (Barrouin‐Melo et al. 2016). One or more authors were employed by the pet food producers Hill's Pet Nutrition Inc. (Hall et al. 2011, 2002; Wander et al. 1997; Jackson and Jewell 2023), Mark Morris Associates (Brown et al. 1998, 2000), Royal Canin (Smith et al. 2007) or the Iams Company (Kearns et al. 1999). For the home‐living dogs, there was a risk of the owners not complying with the instructions regarding the amount of feed provided for the dogs, providing the dogs with other foods, and possible use of treats for comfort and reward. Based on these considerations, we graded all articles as having an unclear risk of bias.

To summarise the risk of bias, the included articles were scored with between two and five ‘Yes’, five of the articles were scored with one ‘No’ each, with the rest of the scores being ‘Unclear’. Thus, few of the entries in the SYRCLE's tool were addressed and we conclude that the risk of bias was unclear for the included articles.

### 3.3 Assessments of Quality of Evidence

The quality of evidence for the primary outcomes was evaluated for the included articles (Table S2). All articles were peer‐reviewed (#1), and compliance with animal welfare regulations (#9) was declared in all except three articles (Kearns et al. 1999; Landymore et al. 1985, 1986). Detailed information on the dog breeds used (#2) was described in seven articles (LeBlanc et al. 2005; Hall et al. 2011, 2002; Boretti et al. 2019; Wander et al. 1997; Kearns et al. 1999; Smith et al. 2007), and the sex of the dogs (#3) were stated in all except four of the articles (Kearns et al. 1999; Landymore et al. 1985, 1986; Smith et al. 2007). The housing conditions (#4) were inadequately described for the dogs that were living with their owners, as none of the articles provided information on the home environments of the dogs and the level of physical activity was described in only one article (Barrouin‐Melo et al. 2016). For the dogs that were housed in a research facility, six of the articles described if the dogs were housed individually (LeBlanc et al. 2005; Brown et al. 1998, 2000), in pairs (Hall et al. 2011; Jackson and Jewell 2023) or in groups of four dogs (Boretti et al. 2019), and only two of the articles described social and physical enrichment measures for the dogs (Hall et al. 2011; Jackson and Jewell 2023). Descriptions on when blood was sampled (#5) were included in all articles, and the prandial status of the dogs (#5) were described in all except three articles (Kearns et al. 1999; Landymore et al. 1985, 1986). The source of the marine ingredients tested (#6) was described as cod‐liver oil (Landymore et al. 1985, 1986), ‘fish oil’ (Hall et al. 2011; Pellegrino et al. 2021; Wander et al. 1997; Kearns et al. 1999; Smith et al. 2007), MEG‐3 0355TG fish oil from DSM Inc. (Jackson and Jewell 2023), or as oils originating from menhaden (LeBlanc et al. 2005; Hall et al. 2002; Brown et al. 1998, 2000), salmon (Boretti et al. 2019) or deep sea fish (Barrouin‐Melo et al. 2016). The majority of articles gave details on the statistical method(s) used (#7) with the exception of one article (Hall et al. 2011). Descriptive statistics with a measure of variability (#8) for the serum/plasma TC concentration at endpoint for each of the experimental groups was presented in 10 articles (LeBlanc et al. 2005; Hall et al. 2011, 2002; Boretti et al. 2019; Brown et al. 1998, 2000; Wander et al. 1997; Kearns et al. 1999; Landymore et al. 1985, 1986). One article presented means for the serum/plasma TC concentration at endpoint for each of the experimental groups without a measure of variability (Pellegrino et al. 2021), and one article did not describe if the spread was standard deviation or standard error of the mean and were graded with zero points (Barrouin‐Melo et al. 2016). Two articles did not present data for endpoint TC concentration, but reported the change from baseline values and therefore these articles were not included in the meta‐analysis (Smith et al. 2007; Jackson and Jewell 2023).

A statement of potential conflict of interests (#10) was declared in only five articles (Hall et al. 2011; Boretti et al. 2019; Pellegrino et al. 2021; Barrouin‐Melo et al. 2016; Jackson and Jewell 2023). The mean quality score for all included articles was 7.1 (range 4.5−9.5), with an overall medium‐to‐high quality for all articles.

### 3.4 Details on the Design of the Diets

All included studies used an intervention diet containing marine oils and a control diet with no (or low) contents of marine n‐3 LC‐PUFAs. The dietary compositions showed large variations between the studies with regard to both the amount and type of marine fat (Table 1).

Twelve of the included studies were designed with a pre‐study diet, described as being free of fish oil‐derived n‐3 PUFAs (Barrouin‐Melo et al. 2016), containing no EPA and DHA (Boretti et al. 2019), low in n‐3 PUFAs (Hall et al. 2002), 0.01% EPA and 0.00% DHA (Hall et al. 2011), 0.1% EPA and 0.2% DHA (Pellegrino et al. 2021), 0% EPA and 0.02% DHA (Jackson and Jewell 2023), < 0.1 g EPA and 0.1 g DHA per kg diet (LeBlanc et al. 2005), low concentration of n‐3 fatty acid (Wander et al. 1997), high ratio of n‐6:n‐3 (25:1) (Kearns et al. 1999), or did not specify the contents of EPA and DHA in the diets (Brown et al. 1998, 2000). In one study, using dogs living with the owners, the dogs ate their normal diet in the pre‐study period if the intake corresponded to < 1.5 g of total n‐3 PUFAs per day (Smith et al. 2007). Two articles did not describe any use of a pre‐study diet (Landymore et al. 1985, 1986).

Some of the included studies used more than one intervention group, and based on the PICO criteria, we selected the most relevant intervention and control groups, and the latter was in some cases a different control group for the present review than was identified as a control group in the article. This applies to the following articles: Landymore et al. (1986) included two intervention groups; one received cod‐liver oil and the other received dipyridamole, and only the first group is included in the present review. LeBlanc et al. (2005) included two intervention diets; containing fish oil or fish oil and a high dose of vitamin E, and since the vitamin E content was almost six times higher in the latter diet compared to the control diet (with comparable vitamin E content in the control diet and the fish oil diet), we chose to not include the high‐vitamin E diet. The two studies by Brown et al. (1998, 2000) used two experimental diets in addition to the fish oil diet, containing either safflower oil or beef tallow, and here we chose the diet containing vegetable oil as the control diet rather than the diet with beef tallow since we perceive the former diet as more neutral with regard to its effect on the cholesterol metabolism. Wander et al. (1997) used three diets with different n‐3 PUFA contents; low (EPA and DHA below detection limit), medium (EPA+DHA 2.45 g/kg diet) and high (EPA+DHA 5.65 g/kg diet), and we present findings from low dose n‐3 PUFA as the control diet and high dose n‐3 PUFA as the intervention diet. Hall et al. (2002) used a total of six diets; low dose fish oil (EPA and DHA < 0.1 g/kg diet) or high dose fish oil (EPA 1.9 g/kg diet, DHA 2.5 g/kg diet) combined with one of three doses of α‐tocopherol (17, 101 or 447 mg/kg diet), and here we chose to use only the low dose α‐tocopherol diets as these contained the lowest required amount of this vitamin. Hall et al. (2011) tested five diets, four of which added extra lysine and methionine and contained different amounts of fish oil (0%, 0.45%, 0.9% and 1.35%), and for the present systematic review, we chose 0% fish oil as the control diet and the diet with the highest fish oil content as the intervention diet. Smith et al. (2007) tested three diets; containing either sunflower oil, flax oil or fish oil, and we chose to include the diet containing sunflower as the control diet since flax oil contains a very high amount of α‐linolenic acid which can be converted to EPA and DHA, albeit in low amounts in mammals. Jackson and Jewell (2023) included two intervention diets, containing either MEG‐3 0355TG fish oil (DSM Inc., Parsippany, NJ, USA) or triacylglycerols with a high content of medium‐long saturated fatty acids (CAPTEX‐355, ViaChem Inc., Plano, TX, USA), and only dogs receiving the former diet were included in this review. The remaining articles (Boretti et al. 2019; Pellegrino et al. 2021; Barrouin‐Melo et al. 2016; Kearns et al. 1999; Landymore et al. 1985) contained one control group and one intervention group. The contents of EPA, DHA and/or fish oil for the included intervention diets are presented in Table 1.

Six of the articles specified the source of origin of the oils in the intervention diets as being menhaden (LeBlanc et al. 2005; Hall et al. 2002; Brown et al. 1998, 2000), salmon (Boretti et al. 2019) or deep sea fish (Barrouin‐Melo et al. 2016), but did not provide precise information of the fish species. The remaining articles described the source as ‘fish oil’ (Hall et al. 2011; Pellegrino et al. 2021; Wander et al. 1997; Smith et al. 2007), MEG‐3 0355TG fish oil (Jackson and Jewell 2023) or cod liver oil (Landymore et al. 1985, 1986). The processing of the oils was not described in any of the articles. Only two of the articles described which part(s) of the fish the oils originated from; (Landymore et al. 1985, 1986) using cod liver oil. All the included articles except one (Kearns et al. 1999) described the contents of EPA and/or DHA or the sum of n‐3 PUFAs, either as the content in the diets or in the oils or presented as the daily intake in the dogs.

The diets used for comparison contained either corn oil (Hall et al. 2002; Barrouin‐Melo et al. 2016; Wander et al. 1997), safflower oil (Brown et al. 1998, 2000), sunflower oil (LeBlanc et al. 2005), chicken fat (Kearns et al. 1999), pork fat (Jackson and Jewell 2023), an unspecified oil source (Hall et al. 2011; Boretti et al. 2019; Smith et al. 2007) or no supplementation (Pellegrino et al. 2021; Landymore et al. 1985, 1986).

Two of the articles, both by Landymore et al. (1985, 1986) used intervention and control diets with added 2% cholesterol. The cholesterol content in the experimental diets was not measured in any of the articles.

### 3.5 Reported Effects on the Circulating TC, HDL‐C and LDL‐C Concentrations

Of the 12 included articles that presented information regarding the circulating TC concentration at endpoint, six reported that the TC concentration was lower in the intervention group as compared to its control group at end‐point (Hall et al. 2002; Boretti et al. 2019; Brown et al. 1998; Wander et al. 1997; Landymore et al. 1985, 1986), whereas five reported that TC concentration was similar in intervention and control groups (LeBlanc et al. 2005; Hall et al. 2011; Brown et al. 2000; Pellegrino et al. 2021; Barrouin‐Melo et al. 2016). The article by Kearns et al. (1999) found that serum TC concentration was similar between the intervention group and the control group for young dogs, but was higher in the intervention group compared to the control group in old dogs. Two articles compared the change in TC concentration from baseline measurements to endpoint and did not report actual TC concentration (these are not included in the meta‐analysis). Of these, one reported that change in TC concentration over the intervention period was similar between the intervention and control groups (Smith et al. 2007) whereas the other article reported a larger reduction in TC concentration in the intervention group (Jackson and Jewell 2023).

Only four of the studies investigated effects on the concentration of lipoprotein‐bound cholesterol. A lower HDL‐C concentration in the intervention group was reported in three of these articles (Brown et al. 1998; Pellegrino et al. 2021; Wander et al. 1997), whereas one article found no difference between intervention and control groups for HDL‐C concentration (Brown et al. 2000). Only one article reported on LDL‐C, presenting data that the LDL‐C concentration was not affected by fish oil intake (Pellegrino et al. 2021).

Changes in bodyweight may affect the circulation cholesterol concentration, therefore, information on any change in bodyweight during the intervention period is of importance. Three articles reported an increase in bodyweight in both the intervention and the comparator groups (Brown et al. 1998; Landymore et al. 1985, 1986), four articles reported that the bodyweight was not affected in any of the groups (LeBlanc et al. 2005; Hall et al. 2011; Brown et al. 2000; Smith et al. 2007), whereas three articles provided no information regarding the effect of the intervention on the bodyweight (Pellegrino et al. 2021; Barrouin‐Melo et al. 2016; Kearns et al. 1999). Four articles described that the energy intake was adjusted to keep the dogs' bodyweight and/or BCS (Hall et al. 2002; Boretti et al. 2019; Wander et al. 1997; Jackson and Jewell 2023).

### 3.6 Meta‐Analysis

**Figure 2:**
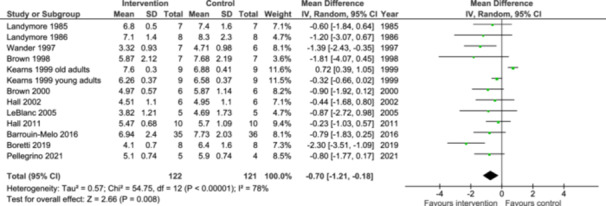
Meta‐analysis using a random effects model presenting the effects of intake of marine oils on circulating total cholesterol concentration (mmol/L) as a forest plot. CI, confidence interval. [Color figure can be viewed at wileyonlinelibrary.com]

Twelve of the identified articles (LeBlanc et al. 2005; Hall et al. 2011, 2002; Boretti et al. 2019; Brown et al. 1998, 2000; Pellegrino et al. 2021; Barrouin‐Melo et al. 2016; Wander et al. 1997; Kearns et al. 1999; Landymore et al. 1985, 1986) were included in the meta‐analysis of the endpoint serum/plasma TC concentration in the intervention and control groups. This comprises the results from 13 studies (two studies in Kearns et al. [1999]) with data from 122 dogs representing intervention groups and 121 dogs representing control groups (Figure 2). The number of dogs in the studies included in the meta‐analysis was between 9 and 71 dogs, with a median of 14 dogs. The result from the meta‐analysis showed that the circulating TC concentration was lower in dogs fed marine oil compared with their control group; the mean difference was −0.70 mmol/L and 95% confidence interval (CI) −1.21, −0.18 mmol/L, with an overall test for effect *Z* = 2.66 and *p* = 0.008. The statistical heterogeneity *χ* ^2^ was 54.75 (*p* < 0.00001) and *I* ^2^ was 78%. Thus, the between‐group differences and the magnitude of effect were highly heterogeneous in our meta‐analysis.

A sensitivity analysis was conducted through a sequential leave‐one‐out analysis of studies with the highest positive and negative effect sizes, and the studies with the highest risk of bias or lowest quality of evidence. Removing the study in old dogs in Kearns et al. (1999) affected the outcome measure considerably, to −0.77 (CI −1.12, −0.42) mmol/L, with *Z* = 4.31, *p* < 0.0001, *χ* ^2^ = 15.46 (*p* = 0.16) and *I* ^2^ = 29%. Other exclusions did not alter the outcome measure (data not presented). Since the statistical heterogeneity was mainly explained by the study in old dogs (Kearns et al. 1999), no subgroup analyses were performed.

The use of a funnel plot is not recommended when the statistical heterogeneity is large (> 50%) (Ioannidis and Trikalinos 2007), as is the case for the present meta‐analysis with a considerable heterogeneity *I* ^2^ of 78%. Taking into consideration that a moderately low number of studies were included in the present meta‐analyses, together with a considerable statistical heterogeneity, we present the funnel plot in Figure S1 without further interpretation.

## 4 Discussion

In this systematic review with meta‐analysis, we aimed to identify and assess all available articles that have investigated the effect of consumption of marine ingredients on the circulating TC concentration in domestic dogs. Through the screening process, we identified studies that tested various marine oils, but no relevant studies using marine organisms other than fish or marine proteins were found. Based on the meta‐analysis of the reported findings in 12 articles with a total of 243 domestic dogs, we present evidence that consuming diets containing marine oils results in a significantly lower TC concentration when compared with their respective control group, with an average lower total TC concentration of −0.70 (CI −1.21, −0.18) mmol/L. To the best of our knowledge, this is the first systematic review and meta‐analysis that explores the reported effects of marine ingredients on the circulating TC concentration in domestic dogs.

The articles that were included in the meta‐analysis show variations in three of the components defined in our PICO strategy; that is, a variety of dog breeds and dogs with different ages (all were adults), sex and health conditions were used, an assortment of marine oils originating from different fish species or other marine sources (not always specified) and different doses were explored in the interventions, and the comparator diets contained different types of vegetable or animal fats (not always specified). All the included articles in the meta‐analysis reported the fourth PICO component, that is, the effects of the intervention on the concentration of TC in serum or plasma. The high statistical heterogeneity in the meta‐analysis with an *I* ^2^ of 78%, was largely due to the data from Kearns et al. (1999) on old dogs, as excluding these data from the meta‐analysis returned an *I* ^2^ of 29% with *p* < 0.0001 for the overall effect. This study by Kearns et al. (1999) is the only that reported a higher TC concentration in the intervention group (old dogs). We have no explanation as to why these data from Kearns et al. (1999) deviate from the other included studies, however, this is the only article that provided no information on the dietary contents of EPA and DHA, the health status of the dogs, or on the marine specie(s) from which the oil was extracted. The lower TC concentration in the meta‐analysis was probably not a result of reduced adiposity in the dogs, as the articles reported either an increase in bodyweight (Brown et al. 1998; Landymore et al. 1985, 1986), no change in bodyweight (LeBlanc et al. 2005; Hall et al. 2011; Brown et al. 2000; Smith et al. 2007) or that the energy intake was adjusted to keep the dogs' bodyweight and/or BCS (Hall et al. 2002; Boretti et al. 2019; Wander et al. 1997; Jackson and Jewell 2023), whereas three articles provided no information on any change in bodyweight (Pellegrino et al. 2021; Barrouin‐Melo et al. 2016; Kearns et al. 1999). None of the included articles provided any explanation for the observed effect of marine oils on the TC concentration. Since elevated TC is a risk factor for some diseases in dogs such as atherosclerosis and osteoarthritis (Mahley et al. 1977; Geer 1965; Liu et al. 1986; Kagawa et al. 1998; Boynosky and Stokking 2014; Sottiaux 1999; Hess, Kass, and Winkle 2003; Hess, Kass, and Van Winkle 2006; Saberianpour et al. 2022), the average lower total TC concentration of −0.70 (CI −1.21, −0.18) mmol/L in dogs fed marine oils may have clinical importance and should be further investigated in long‐term studies.

All included articles were assessed for risk of bias, but since the majority of the entries in the SYRCLE's risk of bias tool (Hooijmans et al. 2014) were not addressed in the articles, the risk of bias for the studies was difficult to assess and were therefore graded as having an unclear risk of bias to the reported findings. Especially the last item in the SYRCLE tool, that is, ‘Other sources of bias’ was taken into consideration. Eleven of the 14 articles stated that the studies were supported by the pet food industry, either through the employment of one or more of the authors, through (partial) funding of the project, or by donating the feed for the study, and these associations to the industry may, consciously or unconsciously, influence the design and interpretation of the studies. Another point of concern was the lack of information regarding the housing conditions for dogs living with their owners, and the lack of information concerning the guidelines for use of treats and nonexperimental feeds as well as physical activity for these dogs. The overall quality of the studies was evaluated using the CAMARADES checklist (Macleod et al. 2004) and the ARRIVE 2.0 guidelines (Percie du Sert et al. 2020) and was considered to be of high quality with regard to the primary aim of the present systematic review and meta‐analysis.

## 5 Strength and Limitations

A strength to the interpretation of the lower TC concentration in adult dogs fed diets containing n‐3 LC‐PUFAs, is that despite the heterogeneity in designs with regard to breeds, health status, type and dose of marine oil, duration and the macronutrient compositions of the diets, the result of the meta‐analysis is statistically significant even with the low number of included studies.

There are some limitations to the interpretation of the present systematic review and the meta‐analysis that should be considered; especially the limited number of articles and the lack of homogeneity of the study design in the included articles. A major limitation in relation to our aim to investigate the effects of marine ingredients on TC concentration in dogs is that we did not identify any relevant high‐quality articles using marine ingredients other than oil. A limitation to the interpretation of the meta‐analysis is that most of the dogs included had TC concentration within the normal reference range at baseline, although based on the stated mean and spread of data, some of the dogs had elevated TC concentration (Barrouin‐Melo et al. 2016; Smith et al. 2007). Addition of cholesterol to the diets resulted in above‐normal circulating TC concentration at endpoint, with a less prominent increase in the intervention group relative to the comparator groups (Landymore et al. 1985, 1986). Dogs that had undergone a partial nephrectomy also had elevated circulating TC concentration after 20 months in the study by Brown et al. (1998), in line with a study from the same research group showing that hypercholesterolaemia is directly related to the rate of deterioration of kidney function after partial nephrectomy in dogs (Brown et al. 1991). In the study by Brown et al. (2000) with a shorter duration of 10−13 weeks, the increase in TC concentration after partial nephrectomy was less pronounced. The serum TC concentration was also elevated in the study by Barrouin‐Melo et al. (2016), who used dogs with canine spontaneous osteoarthritis, for which elevated TC concentration is a risk factor (Saberianpour et al. 2022). Thus, the effect of marine ingredients in hypercholesterolemic dogs is still unknown. Another limitation of the present study is that of the included articles, the effect of marine ingredients on circulating cholesterol concentration was the primary aim only in the study by Brown et al. (2000) Also, the lack of information on the dietary compositions of the intervention and comparator diets in two of the articles (Boretti et al. 2019; Smith et al. 2007) limit the interpretation of the impact of the marine ingredients on TC concentration.

## 6 Conclusion

We did not identify any studies using marine organisms other than fish or marine proteins, and the included studies and, thus, the meta‐analysis were therefore limited to marine oils as the intervention. Based on the results provided from the meta‐analysis, there is evidence that consuming marine oil results in a lower circulating TC concentration in domestic dogs, however, due to the lack of relevant studies, the effect of marine oil on hypercholesterolemic dogs is still unknown. We consider the quality of the included studies to be high but with an uncertain risk of bias.

## Author Contributions

O.A.G. planned and designed the study. All authors conducted the literature search. L.V.A., A.S.F. and O.A.G. conducted the meta‐analysis. O.A.G. drafted the paper and had primary responsibility for the final content, and all authors contributed to the writing and approved the final version of the manuscript.

## Animal Welfare Statement

This manuscript does not include original research data.

## Conflicts of Interest

The authors declare no conflicts of interest.

## Supporting information

Supporting information Figure S1: Funnel plot showing the effect estimate with 95% CIs for the effect of intake of diets containing marine oil on circulating total cholesterol concentration.

**Supporting information Table S1:** Evaluation of the risk of bias for the included articles (Yes; low risk of bias, No; high risk of bias, Unclear; Unclear risk of bias).

**Supporting information Table S2:** Study quality checklist for the included studies. Studies were scored with maximum one point per item when the information was provided and with zero points if the information was missing.

## Data Availability

The data presented in this study are available upon request from the corresponding author.
