# Supplementary figures and images for: Effects of Dietary Intake of Marine Ingredients on the Circulating Total Cholesterol Concentration in Domestic Dogs: A Systematic Review and Meta‐Analysis
Source: J Anim Physiol Anim Nutr (Berl). 2024 Sep 18;109(1):183–202. doi: 10.1111/jpn.14045 (PMC11731426; doi:10.1111/jpn.14045)

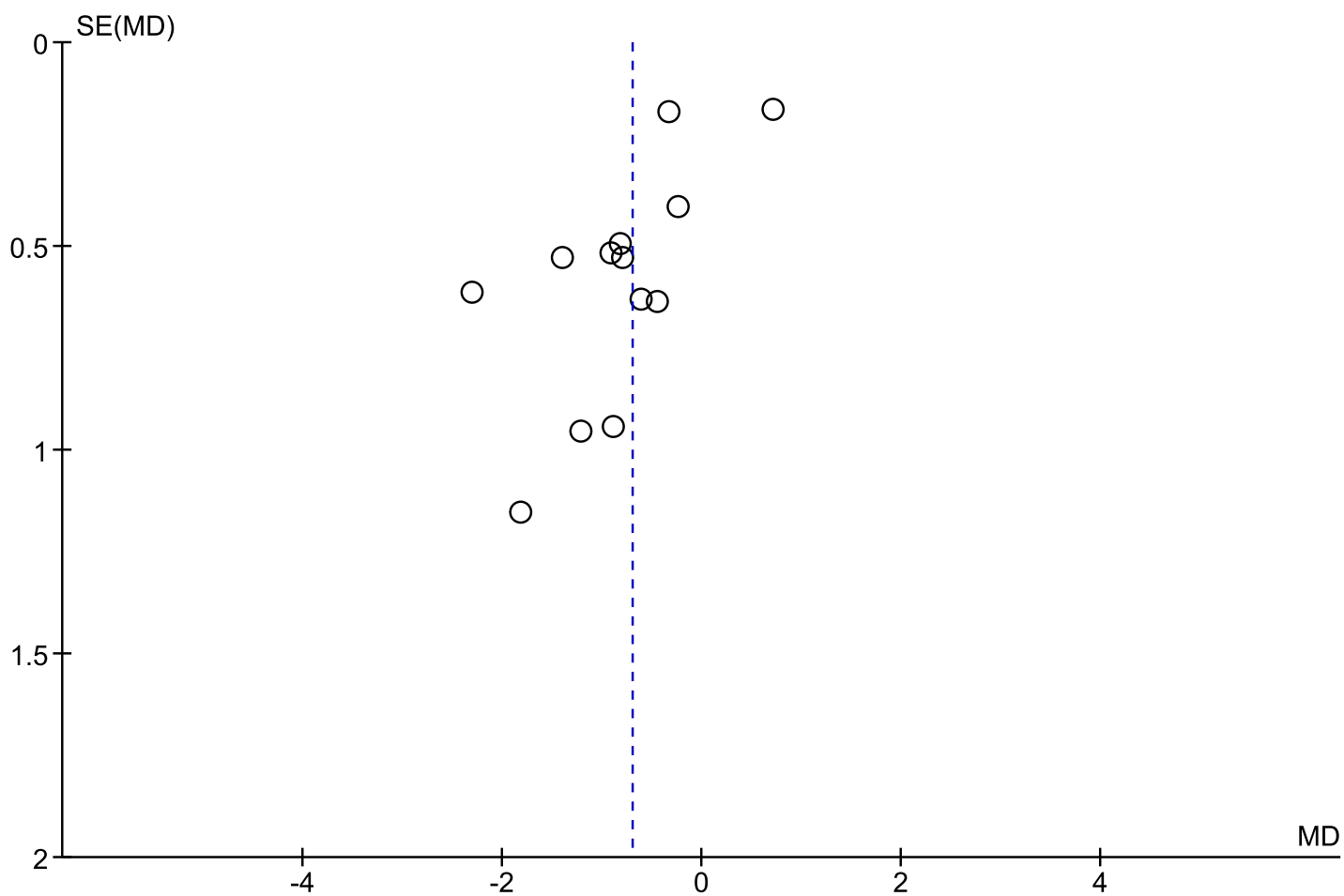

Supplement: Supplementary file 1 — Supporting information Figure S1: Funnel plot showing the effect estimate with 95% CIs for the effect of intake of diets containing marine oil on circulating total cholesterol concentration. [file JPN-109-183-s002.pdf]
