# Supplementary material for: Effects of Dietary Intake of Marine Ingredients on the Circulating Total Cholesterol Concentration in Domestic Dogs: A Systematic Review and Meta‐Analysis
Source: J Anim Physiol Anim Nutr (Berl). 2024 Sep 18;109(1):183–202. doi: 10.1111/jpn.14045 (PMC11731426; doi:10.1111/jpn.14045)
Supplement: Supplementary file 2 — Supporting information Table S1: Evaluation of the risk of bias for the included articles (Yes; low risk of bias, No; high risk of bias, Unclear; Unclear risk of bias). [file JPN-109-183-s003.docx]

**Supplemental table 1:** Evaluation of the risk of bias for the included articles (Yes; low risk of bias, No; high risk of bias, Unclear; Unclear risk of bias)

|  | SYRCLES items with signalling questions | | | | | | | | | |
| --- | --- | --- | --- | --- | --- | --- | --- | --- | --- | --- |
|  | 1. Sequence generation (selection bias) | 2. Baseline characteristics (selection bias) | 3. Allocation concealment (selection bias) | 4. Random housing (performance bias) | 5. Blinding (performance bias) | 6. Random outcome assessment (detection bias) | 7. Blinding (detection bias) | 8. Incomplete outcome data (attrition bias)* | 9. Selective outcome reporting (reporting bias) | 10. Other sources of bias |
|  | Was the allocation sequence adequately generated and applied? | Were the groups similar at baseline, or were they adjusted for confounders in the analysis? | Was the allocation adequately concealed? | Were the animals randomly housed during the experiment? | Were the caregivers and/or investigators blinded from knowledge which intervention each animal received during the experiment? | Were animals selected at random for outcome assessment? | Was the outcome assessor blinded? | Were incomplete outcome data adequately addressed? | Are reports of the study free of selective outcome reporting? | Was the study apparently free of other problems that could result in a high risk of bias? |
| ([Landymore, Kinley et al. 1985](#_ENREF_9)) | Unclear | Unclear | Unclear | Yes | No | Unclear | Unclear | Yes | Unclear | Unclear |
| ([Landymore, MacAulay et al. 1986](#_ENREF_10)) | Unclear | Unclear | Unclear | Yes | No | Unclear | Unclear | Yes | Unclear | Unclear |
| ([Wander, Hall et al. 1997](#_ENREF_14)) | No | Yes | Unclear | Yes | Unclear | Unclear | Unclear | Yes | Unclear | Unclear |
| ([Brown, Brown et al. 1998](#_ENREF_3)) | Unclear | Yes | Unclear | Yes | Unclear | Unclear | Unclear | Yes | Unclear | Unclear |
| ([Kearns, Hayek et al. 1999](#_ENREF_8)) | Unclear | Unclear | Unclear | Yes | Unclear | Unclear | Unclear | Yes | Unclear | Unclear |
| ([Brown, Brown et al. 2000](#_ENREF_4)) | Unclear | Yes | Unclear | Yes | Unclear | Unclear | Unclear | Yes | Unclear | Unclear |
| ([Hall, Tooley et al. 2002](#_ENREF_6)) | No | Yes | Unclear | Yes | Unclear | Unclear | Unclear | Yes | Unclear | Unclear |
| ([LeBlanc, Bauer et al. 2005](#_ENREF_11)) | Unclear | Yes | Unclear | Yes | Unclear | Unclear | Unclear | Yes | Unclear | Unclear |
| ([Smith, Freeman et al. 2007](#_ENREF_13)) | Yes | Yes | Unclear | Yes | Yes | Unclear | Unclear | Yes | Unclear | Unclear |
| ([Hall, Chinn et al. 2011](#_ENREF_5)) | Unclear | Yes | Unclear | Yes | Unclear | Unclear | Unclear | Yes | Unclear | Unclear |
| ([Barrouin-Melo, Anturaniemi et al. 2016](#_ENREF_1)) | Yes | Unclear | Unclear | Yes | Yes | Unclear | Yes | Yes | Unclear | Unclear |
| ([Boretti, Burla et al. 2019](#_ENREF_2)) | Unclear | Yes | Unclear | Yes | Unclear | Unclear | Unclear | Yes | Unclear | Unclear |
| ([Pellegrino, Risso et al. 2021](#_ENREF_12)) | Yes | Unclear | Unclear | Yes | No | Unclear | Yes | Yes | Unclear | Unclear |
| ([Jackson and Jewell 2023](#_ENREF_7)) | Unclear | Yes | Unclear | Yes | Yes | Unclear | Yes | Yes | Unclear | Unclear |

*For the main outcome in the present systematic review and meta-analysis, i.e., serum/plasma total cholesterol concentration

**References**

Barrouin-Melo, S. M., J. Anturaniemi, S. Sankari, M. Griinari, F. Atroshi, S. Ounjaijean and A. K. Hielm-Bjorkman (2016). "Evaluating oxidative stress, serological- and haematological status of dogs suffering from osteoarthritis, after supplementing their diet with fish or corn oil." Lipids in Health and Disease **15**: 139-156.

Boretti, F. S., B. Burla, J. Deuel, L. Gao, M. R. Wenk, A. Liesegang and N. S. Sieber-Ruckstuhl (2019). "Serum lipidome analysis of healthy beagle dogs receiving different diets." Metabolomics **16**(1): 1-12.

Brown, S. A., C. A. Brown, W. A. Crowell, J. A. Barsanti, T. Allen, C. Cowell and D. R. Finco (1998). "Beneficial effects of chronic administration of dietary omega-3 polyunsaturated fatty acids in dogs with renal insufficiency." J Lab Clin Med **131**(5): 447-455.

Brown, S. A., C. A. Brown, W. A. Crowell, J. A. Barsanti, C. W. Kang, T. Allen, C. Cowell and D. R. Finco (2000). "Effects of dietary polyunsaturated fatty acid supplementation in early renal insufficiency in dogs." J Lab Clin Med **135**(3): 275-286.

Hall, J. A., R. M. Chinn, W. R. Vorachek, M. E. Gorman, J. L. Greitl, D. K. Joshi and D. E. Jewell (2011). "Influence of dietary antioxidants and fatty acids on neutrophil mediated bacterial killing and gene expression in healthy Beagles." Veterinary Immunology and Immunopathology **139**(2-4): 217-228.

Hall, J. A., K. A. Tooley, J. L. Gradin, D. E. Jewell and R. C. Wander (2002). "Influence of dietary long-chain n-3 fatty acids from Menhaden fish oil on plasma concentrations of alpha-tocopherol in geriatric Beagles." American Journal of Veterinary Research **63**(1): 104-110.

Jackson, M. I. and D. E. Jewell (2023). "Feeding of fish oil and medium-chain triglycerides to canines impacts circulating structural and energetic lipids, endocannabinoids, and non-lipid metabolite profiles." Front Vet Sci **10**: 1-18.

Kearns, R. J., M. G. Hayek, J. J. Turek, M. Meydani, J. R. Burr, R. J. Greene, C. A. Marshall, S. M. Adams, R. C. Borgert and G. A. Reinhart (1999). "Effect of age, breed and dietary omega-6 (n-6): omega-3 (n-3) fatty acid ratio on immune function, eicosanoid production, and lipid peroxidation in young and aged dogs." Vet Immunol Immunopathol **69**(2-4): 165-183.

Landymore, R. W., C. E. Kinley, J. H. Cooper, M. MacAulay, B. Sheridan and C. Cameron (1985). "Cod-liver oil in the prevention of intimal hyperplasia in autogenous vein grafts used for arterial bypass." J Thorac Cardiovasc Surg **89**(3): 351-357.

Landymore, R. W., M. MacAulay, B. Sheridan and C. Cameron (1986). "Comparison of cod-liver oil and aspirin-dipyridamole for the prevention of intimal hyperplasia in autologous vein grafts." Ann Thorac Surg **41**(1): 54-57.

LeBlanc, C. J., J. E. Bauer, G. Hosgood and G. E. Mauldin (2005). "Effect of dietary fish oil and vitamin E supplementation on hematologic and serum biochemical analytes and oxidative status in young dogs." Vet Ther **6**(4): 325-340.

Pellegrino, F. J., A. Risso, Y. Corrada, R. C. Gambaro and A. I. Seoane (2021). "Influence of dietary fish oil supplementation on DNA damage in peripheral blood lymphocytes of nine healthy dogs." Veterinary Record Open **8**(1): e12-e16.

Smith, C. E., L. M. Freeman, J. E. Rush, S. M. Cunningham and V. Biourge (2007). "Omega-3 fatty acids in Boxer dogs with arrhythmogenic right ventricular cardiomyopathy." J Vet Intern Med **21**(2): 265-273.

Wander, R. C., J. A. Hall, J. L. Gradin, S. H. Du and D. E. Jewell (1997). "The ratio of dietary (n-6) to (n-3) fatty acids influences immune system function, eicosanoid metabolism, lipid peroxidation and vitamin E status in aged dogs." J Nutr **127**(6): 1198-1205.
