# Supplementary material for: Effects of Dietary Intake of Marine Ingredients on the Circulating Total Cholesterol Concentration in Domestic Dogs: A Systematic Review and Meta‐Analysis
Source: J Anim Physiol Anim Nutr (Berl). 2024 Sep 18;109(1):183–202. doi: 10.1111/jpn.14045 (PMC11731426; doi:10.1111/jpn.14045)
Supplement: Supplementary file 3 — Supporting information Table S2: Study quality checklist for the included studies. Studies were scored with maximum one point per item when the information was provided and with zero points if the information was missing. [file JPN-109-183-s001.docx]

**Supplemental Table 2:** Study quality checklist for the included studies. Studies were scored with maximum one point per item when the information was provided and with zero points if the information was missing.

| Refs. | 1. Peer reviewed publication | 2. Breeds | 3. Sex of the experimental animals | 4. Housing conditions (dogs per cage), any actions to improve animal welfare of the experimental dogs including level of physical activity | 5. Description of the procedures, including when it was done, any preparation of the dogs before blood sampling, and prandial status | 6. Source of the marine ingredient tested | 7. Details of the statistical methods used for each analysis | 8. Summary/descriptive statistics for each experimental group, with a measure of variability where applicable | 9. Compliance with animal welfare regulations | 10. Statement of potential conflict of interests | Total score (of 10) |
| --- | --- | --- | --- | --- | --- | --- | --- | --- | --- | --- | --- |
| ([Landymore, Kinley et al. 1985](#_ENREF_10)) | 1 | 0 | 0 | 0 | 0.5^§^ | 1 | 1 | 1 | 0 | 0 | 4.5 |
| ([Landymore, MacAulay et al. 1986](#_ENREF_11)) | 1 | 0 | 0 | 0 | 0.5^§^ | 1 | 1 | 1 | 0 | 0 | 4.5 |
| ([Wander, Hall et al. 1997](#_ENREF_15)) | 1 | 1 | 1 | 0^†‡^ | 1 | 0.5^\|\|^ | 1 | 1 | 1 | 0 | 7.5 |
| ([Brown, Brown et al. 1998](#_ENREF_3)) | 1 | 0 | 1 | 0.5^‡^ | 1 | 1 | 1 | 1 | 1 | 0 | 7.5 |
| ([Kearns, Hayek et al. 1999](#_ENREF_9)) | 1 | 1 | 0 | 0^†‡^ | 0.5^§^ | 0.5^\|\|^ | 1 | 1 | 0 | 0 | 5.0 |
| ([Brown, Brown et al. 2000](#_ENREF_4)) | 1 | 0 | 1 | 0.5^‡^ | 1 | 1 | 1 | 1 | 1 | 0 | 7.5 |
| ([Hall, Tooley et al. 2002](#_ENREF_6)) | 1 | 1 | 1 | 0^†‡^ | 1 | 1 | 1 | 1 | 1 | 0 | 8.0 |
| ([LeBlanc, Bauer et al. 2005](#_ENREF_12)) | 1 | 1 | 1 | 0.5^‡^ | 1 | 1 | 1 | 1 | 1 | 0 | 8.5 |
| ([Smith, Freeman et al. 2007](#_ENREF_14)) | 1 | 1 | 0 | 0^†‡^ | 1 | 0.5^\|\|^ | 1 | 0 | 1 | 0 | 5.5 |
| ([Hall, Chinn et al. 2011](#_ENREF_5)) | 1 | 1 | 1 | 1 | 1 | 0.5^\|\|^ | 0 | 1 | 1 | 1 | 8.5 |
| ([Barrouin-Melo, Anturaniemi et al. 2016](#_ENREF_1)) | 1 | 0.5^*¶^ | 1^¶^ | 0.5^†^ | 1 | 0.5^\|\|^ | 1 | 0 | 1 | 1 | 7.5 |
| ([Boretti, Burla et al. 2019](#_ENREF_2)) | 1 | 1 | 1 | 0.5^‡^ | 1 | 1 | 1 | 1 | 1 | 1 | 9.5 |
| ([Pellegrino, Risso et al. 2021](#_ENREF_13)) | 1 | 0.5^*^ | 1 | 0^†‡^ | 1 | 0.5^\|\|^ | 1 | 0 | 1 | 1 | 7.0 |
| ([Jackson and Jewell 2023](#_ENREF_8)) | 1 | 0.5^*^ | 1 | 1 | 1 | 0.5^\|\|^ | 1 | 0 | 1 | 1 | 8.0 |

Comments to the grading of some of the items in the checklist:

^*^information on the pure breed dogs were provided, but information on the mixed-breed dogs was not provided (0.5 point subtracted)

^†^information on the number of dogs housed together was not provided (0.5 point subtracted)

^‡^information on actions to improve animal welfare was not provided (0.5 point subtracted)

^§^information on fasting condition at blood sampling was not provided (0.5 point subtracted)

^||^information on the fish specie from which the marine oil originated was not provided (0.5 point subtracted)

^¶^information from Hielm-Björkman *et al.* ([Hielm-Björkman, Roine et al. 2012](#_ENREF_7))

**References**

Barrouin-Melo, S. M., J. Anturaniemi, S. Sankari, M. Griinari, F. Atroshi, S. Ounjaijean and A. K. Hielm-Bjorkman (2016). "Evaluating oxidative stress, serological- and haematological status of dogs suffering from osteoarthritis, after supplementing their diet with fish or corn oil." Lipids in Health and Disease **15**: 139-156.

Boretti, F. S., B. Burla, J. Deuel, L. Gao, M. R. Wenk, A. Liesegang and N. S. Sieber-Ruckstuhl (2019). "Serum lipidome analysis of healthy beagle dogs receiving different diets." Metabolomics **16**(1): 1-12.

Brown, S. A., C. A. Brown, W. A. Crowell, J. A. Barsanti, T. Allen, C. Cowell and D. R. Finco (1998). "Beneficial effects of chronic administration of dietary omega-3 polyunsaturated fatty acids in dogs with renal insufficiency." J Lab Clin Med **131**(5): 447-455.

Brown, S. A., C. A. Brown, W. A. Crowell, J. A. Barsanti, C. W. Kang, T. Allen, C. Cowell and D. R. Finco (2000). "Effects of dietary polyunsaturated fatty acid supplementation in early renal insufficiency in dogs." J Lab Clin Med **135**(3): 275-286.

Hall, J. A., R. M. Chinn, W. R. Vorachek, M. E. Gorman, J. L. Greitl, D. K. Joshi and D. E. Jewell (2011). "Influence of dietary antioxidants and fatty acids on neutrophil mediated bacterial killing and gene expression in healthy Beagles." Veterinary Immunology and Immunopathology **139**(2-4): 217-228.

Hall, J. A., K. A. Tooley, J. L. Gradin, D. E. Jewell and R. C. Wander (2002). "Influence of dietary long-chain n-3 fatty acids from Menhaden fish oil on plasma concentrations of alpha-tocopherol in geriatric Beagles." American Journal of Veterinary Research **63**(1): 104-110.

Hielm-Björkman, A., J. Roine, K. Elo, A. Lappalainen, J. Junnila and O. Laitinen-Vapaavuori (2012). "An un-commissioned randomized, placebo-controlled double-blind study to test the effect of deep sea fish oil as a pain reliever for dogs suffering from canine OA." Bmc Veterinary Research **8**: 157-171.

Jackson, M. I. and D. E. Jewell (2023). "Feeding of fish oil and medium-chain triglycerides to canines impacts circulating structural and energetic lipids, endocannabinoids, and non-lipid metabolite profiles." Front Vet Sci **10**: 1-18.

Kearns, R. J., M. G. Hayek, J. J. Turek, M. Meydani, J. R. Burr, R. J. Greene, C. A. Marshall, S. M. Adams, R. C. Borgert and G. A. Reinhart (1999). "Effect of age, breed and dietary omega-6 (n-6): omega-3 (n-3) fatty acid ratio on immune function, eicosanoid production, and lipid peroxidation in young and aged dogs." Vet Immunol Immunopathol **69**(2-4): 165-183.

Landymore, R. W., C. E. Kinley, J. H. Cooper, M. MacAulay, B. Sheridan and C. Cameron (1985). "Cod-liver oil in the prevention of intimal hyperplasia in autogenous vein grafts used for arterial bypass." J Thorac Cardiovasc Surg **89**(3): 351-357.

Landymore, R. W., M. MacAulay, B. Sheridan and C. Cameron (1986). "Comparison of cod-liver oil and aspirin-dipyridamole for the prevention of intimal hyperplasia in autologous vein grafts." Ann Thorac Surg **41**(1): 54-57.

LeBlanc, C. J., J. E. Bauer, G. Hosgood and G. E. Mauldin (2005). "Effect of dietary fish oil and vitamin E supplementation on hematologic and serum biochemical analytes and oxidative status in young dogs." Vet Ther **6**(4): 325-340.

Pellegrino, F. J., A. Risso, Y. Corrada, R. C. Gambaro and A. I. Seoane (2021). "Influence of dietary fish oil supplementation on DNA damage in peripheral blood lymphocytes of nine healthy dogs." Veterinary Record Open **8**(1): e12-e16.

Smith, C. E., L. M. Freeman, J. E. Rush, S. M. Cunningham and V. Biourge (2007). "Omega-3 fatty acids in Boxer dogs with arrhythmogenic right ventricular cardiomyopathy." J Vet Intern Med **21**(2): 265-273.

Wander, R. C., J. A. Hall, J. L. Gradin, S. H. Du and D. E. Jewell (1997). "The ratio of dietary (n-6) to (n-3) fatty acids influences immune system function, eicosanoid metabolism, lipid peroxidation and vitamin E status in aged dogs." J Nutr **127**(6): 1198-1205.
